# Regulation of Inflammatory Cytokines in Lipopolysaccharide-Stimulated RAW 264.7 Murine Macrophage by 7-*O*-Methyl-naringenin

**DOI:** 10.3390/molecules17033574

**Published:** 2012-03-22

**Authors:** Lanan Wassy Soromou, Zhichao Zhang, Rongtao Li, Na Chen, Weixiao Guo, Meixia Huo, Shuang Guan, Jing Lu, Xuming Deng

**Affiliations:** 1 College of Animal Science and Veterinary Medicine, Jilin University, Changchun 130062, Jilin, China; 2 ChangChun Central Hospital, Changchun 130051, Jilin, China; 3 College of Life Science and Technology, Kunming University of Science and Technology, Kunming 650224, China; 4 Laboratory of Nutrition and Function Food, Jilin University, Changchun 130062, Jilin, China

**Keywords:** 7-*O*-methylnaringenin, anti-inflammation, signal pathways, cytokines

## Abstract

7-*O*-Methylnaringenin, extracted from *Rhododendron speciferum*, belongs to the flavanone class of polyphenols. In the present study, we investigated the anti-inflammatory effects of 7-*O*-methylnaringenin on cytokine production by lipopoly-saccharide (LPS)-stimulated RAW 264.7 macrophages *in vitro*. The results showed that pretreatment with 10, 20 or 40 μg/mL of 7-*O*-methylnaringenin could downregulate tumour necrosis factor (TNF-α), interleukin (IL-6) and interleukin (IL-1β) in a dose-dependent manner. Furthermore, we investigated the signal transduction mechanisms to determine how 7-*O*-methylnaringenin affects RAW 264.7 macrophages. The activation of mitogen-activated protein kinases (MAPK) and IκBα were measured by Western blotting. The data showed that 7-*O*-methylnaringenin could downregulate LPS-induced levels of phosphorylation of ERK1/2, JNK and IκBα. These observations indicated that 7-*O*-methylnaringenin modulated inflammatory cytokine responses by blocking NF-қB, ERK1/2 and JNK/MAPKs activation.

## 1. Introduction

Inflammation is a rapid response of tissue to injury and characterized in the acute phase by increased blood flow and vascular permeability along with the accumulation of fluid, leucocytes, and inflammatory mediators, such as cytokines [1]. LPS is a major constituent of the outer membrane of Gram-negative bacteria. It can activate mononuclear phagocytes (monocytes and macrophages) and other types of cells to secrete TNF-α, IL-6, IL-1β. The excessive production of these cytokines may result in the systemic inflammatory response syndrome (SIRS), severe tissue damage, and septic shock [2,3]. Among the cytokines, TNF-α is thought to be one of the most important mediators of inflammatory diseases. It is elevated in some pathogenic conditions and possesses potential toxic effect that results in hypersensitivity reactions with chronic inflammation [4,5]. IL-6 is a cytokine produced by a number of normal and transformed cells. It is believed to be an endogenous mediator of LPS-induced fever [6,7,8]. IL-1 is a multifunctional cytokine that is responsible for various processes including host defense, inflammation and response to injury. It is produced by many cell types, predominantly by macrophages [9,10]. In recent years, people began to use extracts from natural medicinal plants to prevent and treat inflammatory responses by inhibiting inflammatory cytokines, such as TNF-α, IL-1β and IL-6, and this has become an important area of investigation.

**Figure 1:**
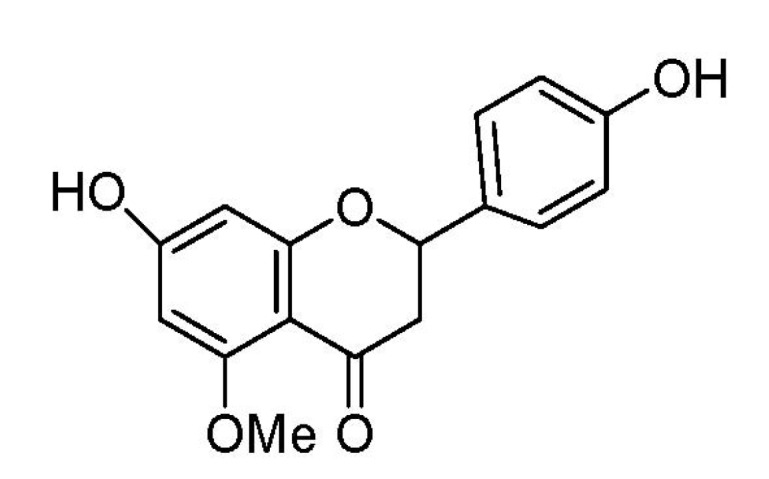
Chemical structure of 7-*O*-methylnaringenin.

Flavones are a group of natural compounds widely generated by plants [11]. Previous studies proved that they have various biological activities, such as anti-oxidant [12], anti-inflammatory [13], anti-allergic [14], anti-infectious [15], anti-carcinogenic [16], pro-apoptotic [17,18] and anti-hypertensive [19] properties. 7-*O*-Methylnaringenin (Figure 1), isolated from *Rhododendron speciferum* for the first time, belongs to the flavanone class of polyphenols [20]. 

Ahmed *et al*. have reported that 7-*O*-methylnaringenin possesses antimalarial activity with a MIC value of approximately 100 µg/mL [21], but there is no description of the anti-inflammatory effects of 7-*O*-methylnaringenin. Thus, the aim of the present study was to investigate the anti-inflammatory effects of 7-*O*-methylnaringenin on LPS-activated pro-inflammatory cytokines production in RAW 264.7 macrophages. Additionally, to determine the mechanism(s) underlying the anti-inflammatory effects of 7-*O*-methylnaringenin, we studied whether this flavone has any influence on the MAPK and NF-κB activation. Our results show that 7-*O*-methylnaringenin significantly inhibits production of pro-inflammatory cytokines induced by LPS *in vitro* by blocking the LPS-induced NF-қB, ERK1/2 and JNK/MAPKs signalling pathways.

## 2. Results and Discussion

### 2.1. Effect of 7-O-Methylnaringenin on Macrophage Toxicity

**Figure 2:**
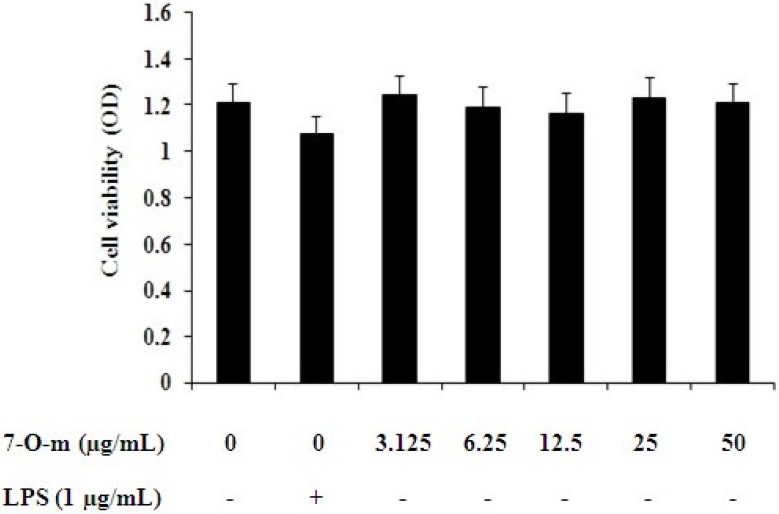
Effects of 7-*O*-methylnaringenin on the viability of RAW 264.7 cells. RAW 264.7 cells were incubated in the presence or absence of 7-*O*-methylnaringenin (0 to 50 μg/mL) for 24 h. Cell viability was determined by the MTT assay. Data are presented as means ± SEM of three independent experiments.

RAW 264.7 cells were incubated with 7-*O*-methylnaringenin in amounts ranging from 0 to 50 μg/mL and cell viability was measured by an MTT assay 18 h later. We found that 7-*O*-methylnaringenin from 0 to 50 μg/mL had no cytotoxic effects on RAW 264.7 cells (Figure 2). These results confirmed that the effects of 7-*O*-methylnaringenin on RAW 264.7 cells were not due to a reduction in cell viability.

### 2.2. Effects of 7-O-Methylnaringenin on LPS-Induced Cytokine Production

**Figure 3:**
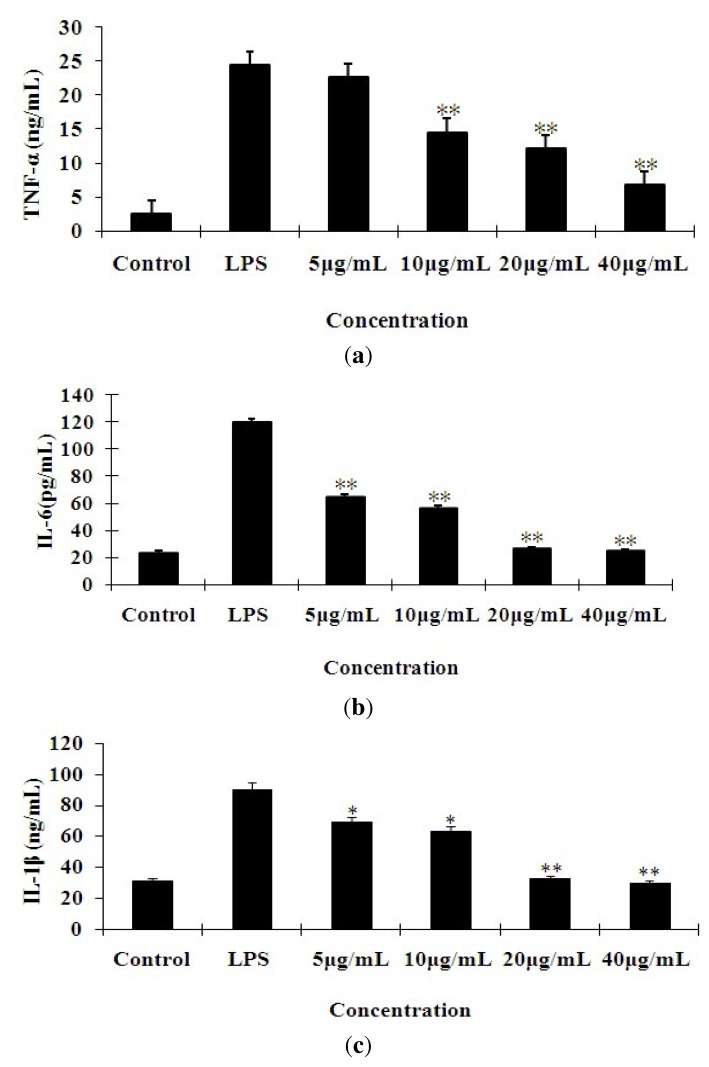
Effect of different concentrations of 7-*O*-methylnaringenin on the secretion of TNF-α (**a**), IL-6 (**b**) and IL-1β (**c**) in LPS-stimulated RAW 264.7 cells. The cells were pretreated with different concentrations (5, 10, 20 and 40 μg/mL) of 7-*O*-methylnaringenin for 1 h prior to stimulation with 1 μg/mL of LPS for 24 h. Control values were obtained in the absence of LPS or 7-*O*-methylnaringenin. The values are means ± SEM of three independent experiments. *p < 0.05, ** *P* < 0.01 *vs*. LPS group.

The concentrations of TNF-α, IL-6 and IL-1β in the culture supernatants of RAW 264.7 cells were measured by an ELISA kit (Figure 3). Treatment of RAW 264.7 cells with LPS alone resulted in significant increases in cytokine production as compared to the control group. The levels of TNF-α, IL-6 and IL-1β were significantly decreased as compared to the LPS group in a dose-dependent manner in 7-*O*-methylnaringenin groups (** *p* < 0.01).

### 2.3. The Effects of 7-O-Methylnaringenin on LPS-Induced MAPK Pathways Activation

**Figure 4:**
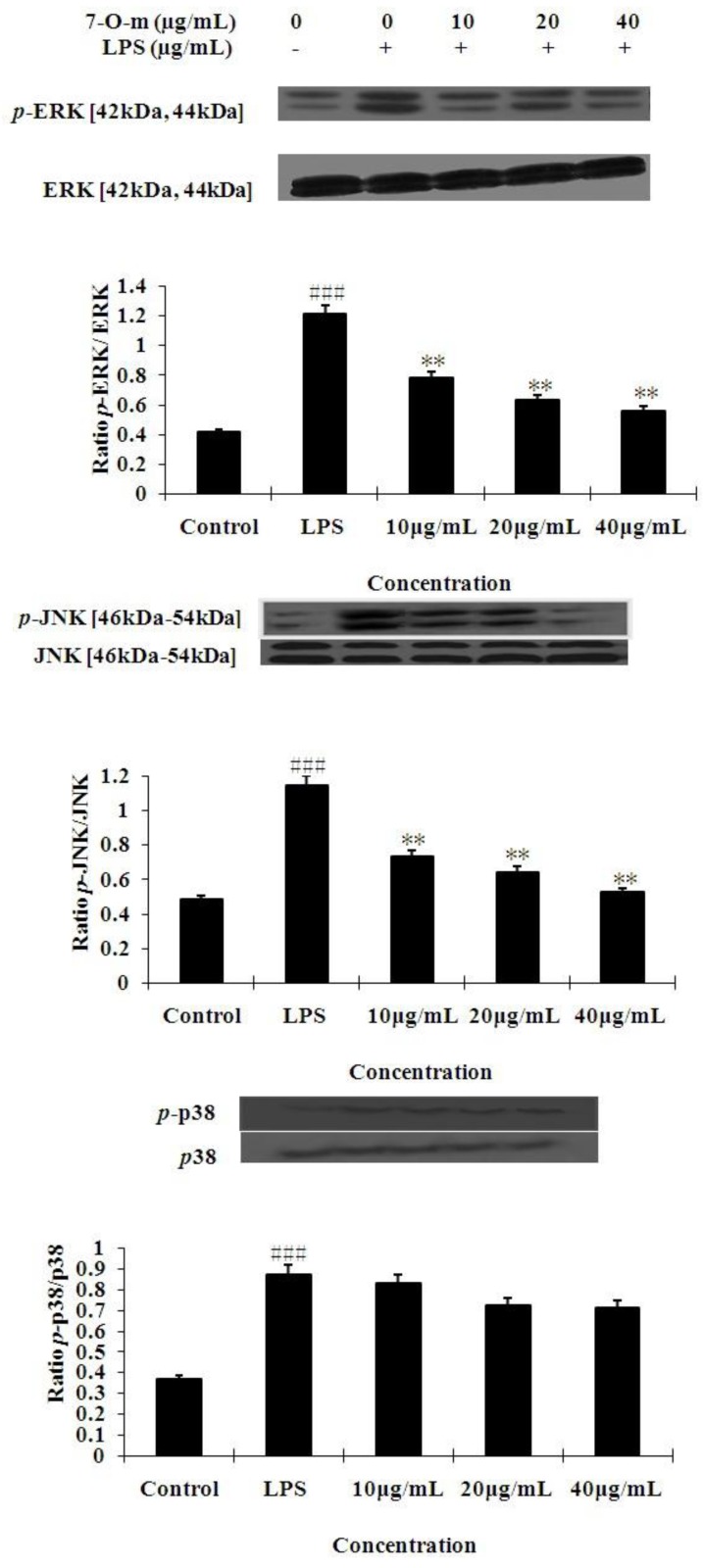
Effects of 7-*O*-methylnaringenin (7-*O*-m) on MAPK in LPS-stimulated RAW 264.7 cells. The cells were pretreated with different concentrations (10, 20, 40 μg/mL) of 7-*O*-methylnaringenin for 1 h and then incubated with or without 1 μg/mL of LPS for 30 min. Protein samples were analyzed by Western blot with phospho-specific antibodies as described in materials and methods. The data are representative of three independent experiments and expressed as mean ± SEM. ** *P* < 0.01 *vs*. LPS group. ^###^ *P* < 0.001 *vs*. control group.

In order to investigate the mechanism by which 7-*O*-methylnaringenin inhibits LPS-induced production of inflammatory cytokines, we examined the effect of 7-*O*-methylnaringenin on the LPS-induced phosphorylation of ERK1/2, JNK and p38 MAPK in the cytoplasm by Western blotting analysis using three different phospho-specific antibodies. As shown in Figure 4, the phosphorylation level of the ERK1/2, JNK and p38 MAPK increased dramatically after 30 min of stimulation with LPS. 7-*O*-methylnaringenin markedly inhibited LPS-induced activation of p-ERK1/2, p-JNK/MAPKs in a dose-dependent manner. But there were no significant changes in p-p38/MAPKs.

### 2.4. The Effect of 7-O-Methylnaringenin on LPS-Induced Degradation and Phosphorylation of IκBα

**Figure 5:**
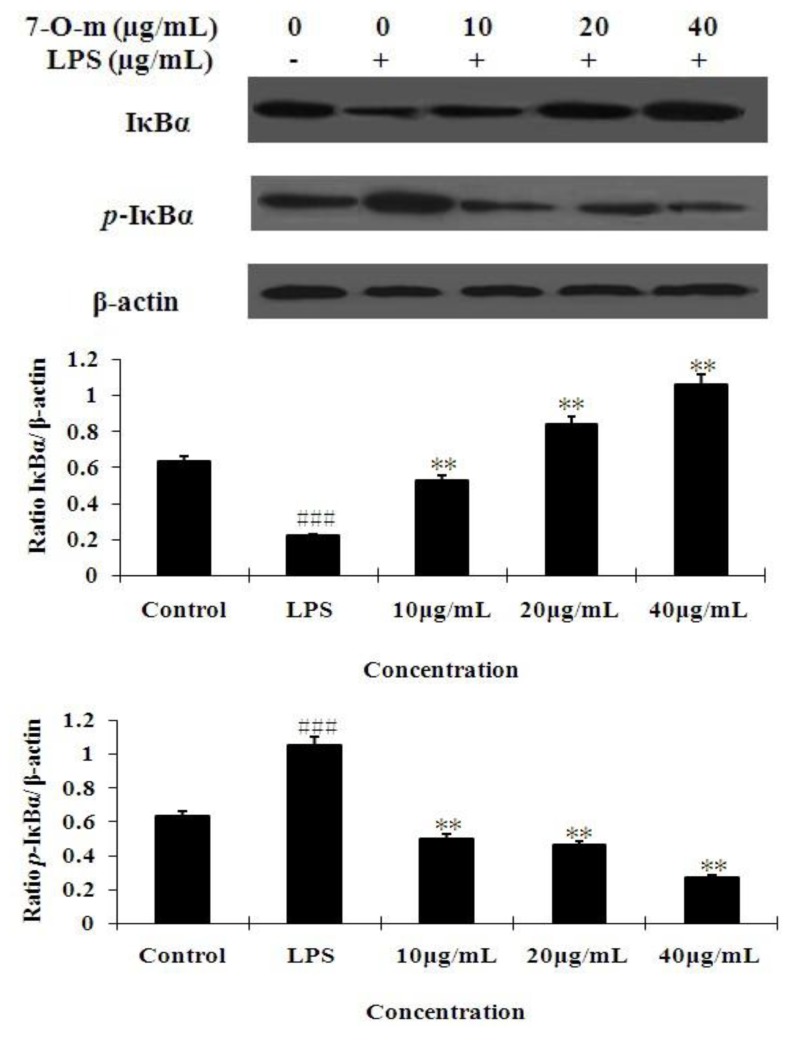
Effect of 7-*O*-methylnaringenin treatment on IκBα phosphorylation and degradation. RAW 264.7 macrophage cells were pretreated with the concentrations (10, 20, 40 μg/mL) of 7-*O*-methylnaringenin for 1 h and then the cells were incubated with LPS (1 mg/L) for 30 min. Total cellular proteins were analyzed by western blot with speciﬁc antibodies. Quantiﬁcation of protein expression was normalized to β-actin using a densitometer(Imaging System). The data are representative of three independentexperiments and expressed as mean ± SEM. ** *P* < 0.01 *vs*. LPS group.^###^ *P* < 0.001 *vs*. control group.

The NF-κB transcription factor is preceded by the degradation and phosphorylation of IκBα. Thus, we examined the effect of 7-*O*-methylnaringenin on IκBα phosphorylation and degradation. Figure 5 shows that LPS-induced IκBα degradation was signiﬁcantly blocked by pre-treatment with 7-*O*-methylnaringenin. To determine whether this IκBα degradation was related to IκBα phosphorylation, we examined the effect of 7-*O*-methylnaringenin on LPS-induced p-IκBα. Western blot analysis showed that 7-*O*-methylnaringenin inhibited LPS-mediated IκBα phosphorylation in a dose-dependent manner.

### 2.5. Discussion

It is well known that pro-inflammatory cytokines induced by LPS, such as TNF-α, IL-6 and IL-1β, play a key role in the process of inflammatory diseases. Excessive production of these pro-inflammatory cytokines will result in a systemic inflammatory response syndrome, such as septic shock. Based on this information, the pharmacological inhibition of these inflammatory mediators is an important target in the treatment of endotoxemia with bacterial infection. In this paper, our data revealed that 7-*O*-methylnaringenin inhibited TNF-α, IL-6 and IL-1β production in a dose-dependent manner in LPS-stimulated RAW 264.7 cells. It should be noted that this inhibitory effect was not due to cytotoxic activity of 7-*O*-methylnaringenin, because the cell viability was not affected by 7-*O*-methylnaringenin treatment.

We also investigated the mechanisms of action underlying such a function. Both MAPKs and NF-κB signaling pathways are involved in the LPS-induced pro-inflammatory mediators and cytokines expression, which play a critical role in the regulation of cell growth and differentiation as well as the control of cellular responses to cytokines and stresses [22,23]. Maximal MAPK expression is known to occur 20–30 min after LPS treatment in human and murine monocytes and macrophages [24]. Inhibition of any of the three MAPK pathways (JNK, p38 MAPK, and ERK) is sufficient to block induction of TNF-α by LPS [25,26,27]. Our results revealed that 7-*O*-methylnaringenin obviously down-regulated LPS-induced phosphorylation of ERK1/2 and JNK pathways in a dose-dependent manner, but the phosphorylation of p38 was not affected by 7-*O*-methylnaringenin treatment in activated macrophage cells (Figure 4). This result suggests that p38 is not involved in the inhibition by 7-*O*-methylnaringenin in RAW 264.7 cells.

Nuclear factor-κB participates in regulating the expression of cytokines and other mediators that are involved in the inflammatory response [28]. Thus, inhibition of the production of these signaling pathways may explain the potent activity of 7-*O*-Methylnaringenin as a suppressor of inflammatory cytokines. In unstimulated condition, NF-κB is located in the cytoplasm as an inactive NF-κB/IκBα complex and its activity is tightly controlled by the inhibitory protein IκBα. The phosphorylation-induced degradation of the IκBα released the NF-κB to enter the nucleus and activate specific target gene expression [29]. Therefore, the activation of NF-κB could be assessed in RAW cells by measuring the degree of phosphorylation of IκBα protein. Incubation of macrophages with LPS caused markedly phosphorylation of cytosolic IκBα, but 7-*O*-methylnaringenin signiﬁcantly inhibited the phosphorylation of IκBα (Figure 5). This means that 7-*O*-methylnaringenin could suppress the activation of the NF-κB signaling pathway.

## 3. Experimental

### 3.1. Materials

7-*O*-Methylnaringenin (purity > 98%) was extracted from *Rhododendron speciferum* as described below. Dimethyl sulfoxide (DMSO), LPS (*Escherichia* *coli* 055:B5), and 3-(4,5-dimethylthiazol-2-y1)-2,5-diphenyltetrazolium bromide (MTT) were purchased from Sigma Chemical Co. (St. Louis, MO, USA). Dulbecco’s modified Eagle’s medium (DMEM), fetal bovine serum (FBS), penicillin and streptomycin for cell culture were obtained from Invitrogen-Gibco (Grand Island, NY, USA). Mouse TNF-α, IL-6 and IL-1β enzyme-linked immunosorbent assay (ELISA) kits were purchased from BioLegend Inc. (San Diego, CA, USA). The rabbit polyclonal anti-p54 JNK, rabbit polyclonal anti-ERK, rabbit polyclonal anti-p38, mouse monoclonal phospho-speciﬁc p46-p54 JNK antibodies, mouse monoclonal anti-phospho-ERK antibodies and mouse monoclonal phospho-speciﬁc p38 antibodies were purchased from Santa Cruz Biotechnology (Santa Cruz, CA, USA). Mouse mAb Phospho-IκBα and rabbit mAb IκBα were purchased from Cell Signaling Technology Inc (Beverly, MA, USA). Peroxidase-conjugated Affinipure goat anti-mouse IgG (H + L) and peroxidase-conjugated Affinipure goat antirabbit IgG (H + L) were purchased from PTG (Chicago, IL, USA).

### 3.2. Preparation of Extract

The air-dried, powdered roots of *Rhododendron speciferum* (9 kg) (collected in Kunming, Yunnan Province, China, in October 2006) were extracted with 75% Me_2_CO/H_2_O (3 × 25 L, 24 h each) at room temperature. The filtrate was concentrated *in vacuo* to give a crude extract, which was then partitioned between H_2_O and EtOAc. The EtOAc portion (250 g) was divided into five fractions (Fractions A–E) over silica gel column (CHCl_3_-MeOH 20:0, 19:1, 9:1, 8:2, 0:20). Fr. B (16 g, CHCl_3_-MeOH 19:1) was then chromatographed on silica gel (CHCl_3_-Me_2_CO 9:1, 8:2, 7:3, 6:4) to afford four subfractions. 7-*O*-Methylnaringenin (20 mg) was obtained from subfraction B1 (CHCl_3_-Me_2_CO 9:1, 2.3 g) by Sephadex LH-20 (CHCl_3_-MeOH 1:1). The compound’s structural identity was determined by one-and two-dimensional nuclear magnetic resonance (NMR) spectroscopic analysis and comparison to published values.

### 3.3. Cell Culture and LPS Stimulation

The RAW 264.7 mouse macrophage cell line was obtained from the China Cell Line Bank (Beijing, China). The cells were cultured in DMEM supplemented with 10% heat-inactivated FBS, 3 mM Glutamine, antibiotics (100 U/mL penicillin and 100 U/mL streptomycin) at 37 °C under a humidified atmosphere of 5% CO_2_. In all experiments, cells were allowed to acclimate for 24 h before any treatments. Cells were incubated with or without 7-*O*-methylnaringenin that was always added 1 h prior to LPS treatment.

### 3.4. MTT Assay for Testing Cell Viability

Cytotoxicity studies induced by 7-*O*-methylnaringenin were performed by the MTT assay. RAW 264.7 cells were mechanically scraped, plated at a density of 4 × 10^5^ cells/mL onto 96-well plates containing 100 μL of DMEM medium, and incubated in a 37 °C, 5% CO_2_ incubator overnight. 7-*O*-methylnaringenin was dissolved in DMSO, and the DMSO concentrations in all assays did not exceed 0.1%. After overnight incubation, the cells were treated with diverse concentrations of 7-*O*-methylnaringenin (0–50 μg/mL) in the presence or absence of LPS (1 μg/mL) according to the experimental design. After 18 h, 20 μL of 5 mg/mL MTT was added to each well and the cells were further incubated for an additional 4 h. MTT was removed and cells were lysed with 150 μL/well DMSO. The optical density was measured at 570 nm on a microplate reader (Tecan, Groedig, Austria).

### 3.5. Measurement of Cytokine Production

To investigate the effect of 7-*O*-methylnaringenin on cytokine levels from LPS-treated cells, RAW 264.7 cells (4 × 10^5^ cells/mL) seeded into 24-well plates were pretreated with 5, 10, 20, 40 μg/mL of 7-*O*-methylnaringenin for 1 h prior to 24 h treatment with 1 mg/L LPS in a 37 °C, 5% CO_2_ incubator. Cell-free supernatants were collected and stored at −20 °C until assayed for cytokine levels. The concentrations of TNF-α, IL-6 and IL-1β in the supernatants of RAW 264.7 cell cultures were determined using an ELISA kit, according to the manufacturer’s instructions (BioLegend, Inc.).

### 3.6. Western Blot Analysis

RAW 264.7 cells (4 × 10^5^ cells/mL) were plated onto 6-well plates and pretreated with 10, 20, 40 μg/mL of 7-*O*-methylnaringenin for 1 h and then stimulated with 1 μg/mL of LPS for 30 min. The cells were collected by centrifugation and washed twice with ice-cold PBS. The washed cell pellets were resuspended in extraction lysis buffer [50 mM Tris (pH 7.6), 150 mM NaCl, 5 mM EDTA (pH 8.0), 0.6% NP-40, 1 mM Na_3_VO_4_, 20 mM β-glycerophosphate, 1 mM phenylmethylsulfonyl fluoride, 2 mM *p*-nitrophenyl phosphate and 1:25 Complete Mini Protease Inhibitor cocktail (Boehringer, Mannheim, Germany)]. After the lysates were incubated on ice for 30 min, they were centrifuged (12,000 g at 4 °C) for 5 min to obtain the cytosolic fraction. The protein concentration was determined using the Bradford assay (Bio-Rad, Munich, Germany) according to the manufacturer’s instructions. The samples of cellular protein from treated and untreated cell extracts were separated by sodium dodecyl sulphate (SDS)-polyacrylamide gel electrophoresis (PAGE) and then electroblotted onto a polyvinylidene difluoride (PVDF) membrane. The immunoblot was incubated overnight with blocking solution (5% (wt/vol) nonfat dry milk), followed by incubation for 4 h with monoclonal phospho-specific antibodies to IκBα, p46–p54 JNK, p42/p44 ERK, and p38mapk in 5% (wt/vol) bovine serum albumin (BSA) dissolved in TTBS. Blots were washed with Tween 20/Tris-buffered saline [TTBS, 20 mM Tris-HCl buffer, pH 7.6, containing 137 mM NaCl and 0.05% (vol/vol) Tween 20] and incubated with a peroxidase-conjugated secondary anti-mouse antibody. Blots were again washed with TTBS and the bound antibodies were detected using ECL plus (GE Healthcare, Buckinghamshire, UK).

### 3.7. Statistical Analysis

Data values were expressed means ± SEM. Data were analyzed by analysis of variance (ANOVA) and two-tailed Student’s t-test. Statistical difference was accepted at *p* < 0.05.

## 4. Conclusions

In summary, the anti-inflammatory effects of 7-*O*-methylnaringenin in LPS-activated macrophages are due to down-regulation the secretion of TNF-α, IL-6 and IL-1β. The related mechanism is by preventing activation of NF-қB, ERK1/2 and JNK/MAPKs signalling pathways. 
